# “Anorexia in a patient with piscosis”

**DOI:** 10.1192/j.eurpsy.2021.960

**Published:** 2021-08-13

**Authors:** C. Capella Meseguer, E. Rodríguez Vázquez, J. Gonçalves Cerejeira, I. Santos Carrasco, A. Gonzaga Ramírez, G. Guerra Valera, M. Queipo De Llano De La Viuda, Ó. Martín

**Affiliations:** 1 Psiquiatría, HCUV, Valladolid, Spain; 2 Psiquiatría, HCUV, VALLADOLID, Spain; 3 Psiquiatría, Hospital Clínico Universitario de Valladolid, Valladolid, Spain

**Keywords:** Anorexia, schizophrénia, eating disorder

## Abstract

**Introduction:**

We present the case of a patient with schizophrenia who presents with restriction of intake, fear of gaining weight and alteration in the way of perceiving herself in which we ask ourselves if these behavioral alterations are secondary to her diagnosis of schizophrenia to an anorexia nervosa independent of previous diagnosis.

**Objectives:**

We propose to carry out a differential diagnosis of alterations in the perception of self-image in a patient with a diagnosis of schizophrenia. We suggest that these alterations may be secondary to alterations in the experience of the self present due to their psychosis.

**Methods:**

In the differential diagnosis of the cause of alterations in self-image and fear of gaining weight, we rely on the psychiatric interview, the study of previous history and different scales: - Eating Disorders Inventory (EDI) - Gardner Body Image Assessment - Weight, body image and self-esteem scale E-PICA - IPASE scale

**Results:**

In this patient in whom the differential diagnosis of the cause of her dietary restrictions and weight loss is proposed, there does not seem to be any psychotic symptoms that produce these alterations.

**Conclusions:**

In the alterations in self-image in those psychotic patients, there is a doubt as to whether these could be secondary to alterations in the perception of the self typical of psychotic diseases or, on the contrary, be secondary to the spectrum of Eating Disorders.

